# Systemic Therapy for Metastatic Pancreatic Cancer

**DOI:** 10.1007/s11864-021-00895-4

**Published:** 2021-10-19

**Authors:** Thomas J. Ettrich, Thomas Seufferlein

**Affiliations:** Department of Internal Medicine I, Ulm University Hospital, Albert-Einstein-Allee 23, 89081 Ulm, Germany

**Keywords:** Pancreatic cancer, Metastatic, Chemotherapy, Systemic therapy, Review

## Abstract

Pancreatic cancer is mainly diagnosed at an advanced, often metastatic stage and still has a poor prognosis. Over the last decades, chemotherapy of metastatic pancreatic cancer (mPDAC) has proven to be superior to a mere supportive treatment with respect to both survival and quality of life. Recently, even sequential treatment of mPDAC could be established. Options for first-line treatment are combination chemotherapy regimens such as FOLFIRINOX and gemcitabine plus nab-paclitaxel when the performance status of the patient is good. For patients with poorer performance status, gemcitabine single-agent treatment is a valid option. Recently, the PARP inhibitor olaparib has been demonstrated to improve progression-free survival when used as a maintenance treatment in the subgroup of patients with mPDAC and a BRCA1/-2 germ line mutation having received at least 16 weeks of platinum-based chemotherapy. This group of patients also benefits from platinum-based chemotherapy combinations. Therefore, the BRCA1/-2 stats should be examined early in patients with mPDAC even when the occurrence of these mutations is only about 5% in the general Caucasian population. After the failure of first-line treatment, patients should be offered a second-line treatment if their ECOG permits further treatment. Here, the combination of 5-FU/FA plus nanoliposomal irinotecan has shown to be superior to 5-FU/FA alone with respect to overall survival. Immune checkpoint inhibitors like PD1/PD-L1 mAbs are particularly efficacious in tumors with high microsatellite instability (MSI-h). Limited data in mPDACs shows that only a part of the already small subgroup of MSI-H mPDACs (frequency about 1%) appears to benefit substantially from a checkpoint inhibitor treatment. The identification of further subgroups, e.g., tumors with DNA damage repair deficiency, gene fusions, as well as novel approaches such as tumor-organoid-informed treatment decisions, may further improve therapeutic efficacy.

## Introduction

Pancreatic cancer still has a dismal prognosis mainly due to the fact that the majority of patients are only diagnosed at an advanced or even metastatic state. Surgery is only appropriate if there is a chance of cure. In contrast to other tumors, surgery of metastasis is not appropriate in pancreatic cancer outside of a clinical trial even in the case of isolated liver metastasis since there is no proof that surgery improves overall survival in this situation [1–5]. Also, radiation therapy plays mainly a supportive role in metastatic disease, e.g., in case of bone metastasis and as a measure to treat tumor-related pain. Therefore, systemic treatment plays an important role in the treatment of metastatic pancreatic cancer (mPDAC). All patients with mPDAC and an ECOG performance status of 0–2 should be offered systemic treatment since it improves both overall survival and quality of life [5–11]. Systemic treatment can also lead to a reduced requirement of pain medication, delay weight loss, as well as the time to the definitive deterioration of quality of life [12]. Systemic treatment should be started immediately after the detection of metastasis.

The performance status of a patient with mPDAC is prognostic. In the case of an ECOG > 2, patients only derive benefit from systemic treatment if the poor performance status is due to the tumor disease itself and is likely to improve if there is tumor remission in response to the treatment. There is also no optimal duration of systemic treatment in mPDAC, and maintenance strategies are only established for specific subgroups of patients. Thus, the duration of treatment in mPDAC depends on its efficacy as well as tolerability and, of course, on the individual patient requirements. Here, we present the current state of systemic treatment of mPDAC.

## First-Line Treatment of mPDAC

There are several well-established options for first-line treatment of mPDAC (see Table 1): The FOLFIRINOX protocol, the combination of gemcitabine plus nab-paclitaxel, and gemcitabine monotherapy. The choice of the respective treatment depends on the patient’s ECOG performance status, comorbidity, and patient’s preferences.

**Table 1:** Metastatic pancreatic ductal adenocarcinoma: palliative first-line regimens

Regiment	Phase	*n*	mPFS (months)	mOS (months)	DCR (%)	References
First-line therapy						
FOLFIRINOX (Prodige-4 Intergroup trial)	III	342	6.4	11.1	70	[6]
Gemcitabine			3.3	6.8	51	
Gemcitabine/Nab-Paclitaxel (MPACT trial)	III	861	5.5	8.7	48	[15]
Gemcitabine			3.7	6.6	33	
Gemcitabine/erlotinib	III	569	3.8	6.2 (RASH 2 + : 10.5)	58	[29, 30]
Gemcitabine			3.6	5.9	49	
Gemcitabine	III	126	2.3	5.7	n.a	[7]
5-FU			0.9	4.4		
Maintenance therapy after first-line therapy						
Olaparib (POLO trial), only pat. with germline BRCA 1 or BRCA 2 mutation and disease control after at least 16 weeks of platinum-based induction therapy	III	154	7.4	19.0	n.a	[31, 32]
Placebo			3.8	19.2		

*mPFS* median progression-free survival, *mOS* median overall survival, *DCR* disease control rate, *FOLFIRINOX* 5-FU, leucovorin, irinotecan, oxaliplatin

### Role of the Performance Status for Treatment Selection

Patients with an ECOG 0–1 benefit from combination chemotherapy, while patients with an ECOG of ≥ 2 and marked comorbidities should preferably receive single-agent treatment. This statement is based on data from a meta-analysis examining gemcitabine or gemcitabine-based combination treatments. Patients with a good performance status (ECOG 0–1) benefitted with respect to survival from combination treatment (HR 0.76). However, when the ECOG was > 1, patients did not benefit from combination treatment (HR 1.08) [13]. There is a limitation to this statement: If tumor-related symptoms are mainly responsible for the poor ECOG and if the ECOG may be improved by tumor remission, combination treatment, e.g., gemcitabine plus nab-paclitaxel, can be given. In this case, lower doses of nab-paclitaxel have been employed [14].

Disregarding the ECOG status, it is paramount that supportive measures such as appropriate pain management and nutritional support after analysis of the nutritional status are also initiated as early as possible.

### Does Age Limit the Treatment Intensity of mPDAC?

There is no sufficient data supporting chronological age as a criterion for the choice of systemic treatment of mPDAC. The biological age of a respective patient appears far more important to decide on the appropriate treatment in the metastatic setting. However, some studies did have an age limit for inclusion of patients into the trial (e.g., the PRODIGE 4 Intergroup study examining FOLFIRINOX vs. gemcitabine only included patients up 75 years of age [6]), and in general, there are few data on patients with advanced age.

## Specific Treatment Protocols

### Gemcitabine Monotherapy

Patients with an ECOG of ≥ 2 or comorbidities that prevent combination treatment should receive a single-agent treatment. Gemcitabine is preferred over 5-FU in this case [7]. There are numerous phase III trials that demonstrate the efficacy of gemcitabine with 1-year overall survival rates of 18–20% and a median OS of about 6 months [6, 15]. Gemcitabine also exhibited a clinical benefit response compared to 5-FU (albeit the latter was used at a suboptimal dose in the trial) [7]. Gemcitabine is generally well tolerated. Its most frequent grade 3/4 side effect is hematotoxicity with leuko-/neutropenia, thrombopenia, and anemia. Other effects such as interstitial pneumonitis are rare, but physicians should be aware of this adverse event to initiate prompt treatment.

### Gemcitabine-Based Combinations

The phase III MPACT trial examined the combination of gemcitabine plus albumin-nanoparticle bound paclitaxel (nab-paclitaxel) compared to gemcitabine single agent. The trial showed a significantly improved mOS compared to the monotherapy (8.5 months compared to 6.7 months, HR 0.72; *p* < 0.001). The combination also significantly improved mPFS (5.5 months vs. 3.7 months, HR 0.69, *p* < 0.001) and response rate (23% vs. 7%, *p* < 0.001). The combination has a higher rate of grade 3/4 side effects with more neutropenia, neuropathy, and diarrhea. The trial recruited patients with a Karnofsky performance status of ≥ 70 so that patients with an ECOG of 0–2 can be treated with this protocol [8, 15, 16]. A phase 2 trial could confirm the safety and efficacy of gemcitabine plus nab-paclitaxel in patients with mPDAC and an ECOG of 2 [14].

Thus, the combination of gemcitabine and nab-paclitaxel can be employed in a larger population of patients with mPDAC (ECOG 0–2) compared to FOLFIRINOX (see below). In addition, the study included elderly patients up to the age of 88 years [15]. The subgroup analysis showed that patients ≥ 65 years of age also benefit from the combination. There is no separate evaluation of the group of patients above 75 years or even 80 years of age from this trial.

MPACT was a study that recruited centers worldwide. The results from the trial could be confirmed in a real-world setting with even better outcome data when only Western countries were considered. For example, mOS was 10.9 months with gemcitabine plus nab-paclitaxel in a retrospective Swedish study [17].

In more recent clinical trials, gemcitabine plus nab-paclitaxel was used as a control arm and also showed good efficiency, for example, an mOS of 11.5 months in phase III HALO-301 trial (gem/nab-paclitaxel vs. gem/nab-paclitaxel + PegPH20 for patients with hyaluronan-high mPDAC) [18]. This could be related to the therapy in further lines, although, especially in this study, patient selection by the inclusion criterion “hyaluronan-high mPDAC” should be considered.

Gemcitabine has been combined with many other drugs such as irinotecan, capecitabine, oxaliplatin, cisplatin, cisplatin/epirubicin, and 5-FU plus docetaxel or exatecan. None of these combinations could show a significant improvement in OS compared to gemcitabine alone in phase III trials in the overall study populations. However, there may be subgroups of patients benefitting from some of these combinations, e.g., patients with an ECOG of 0–1. A significant survival benefit, e.g., of the combination of gemcitabine plus oxaliplatin or cisplatin, could only be demonstrated in meta-analyses. However, the combination of gemcitabine plus cisplatin may be an interesting option for patients with germline BRCA1/2 mutations (see below).

### 5-FU-Based Combinations: FOLFIRINOX

The combination of 5-FU, irinotecan, and oxaliplatin in the FOLFIRINOX protocol is a landmark in the treatment of mPDAC. In the PRODIGE 4 Intergroup trial, this combination treatment achieved a median OS (mOS) of 11.1 months and a median PFS (mPFS) of 6.4 months compared to gemcitabine alone with an mOS of 6.8 months (HR 0.57; *p* < 0.001) and an mPFS of 3.3 months, respectively (HR 0.47; *p* < 0.001) [6]. The data of the initial study could be confirmed in numerous subsequent phase II trials and cohort studies [19].

FOLFIRINOX should be offered to patients with an ECOG of 0–1, a Bilirubin value of ≤ 1.5 × ULN, and a favorable comorbidity profile. This definition comprises about 30% of patients with mPDAC [20]. The toxicity of the FOLFIRINOX protocol is higher than that of gemcitabine with more grade 3/4 neutropenia (45.7% vs. 21%), more febrile neutropenia (5.4% vs. 1.2%), and more diarrhea (12.7% vs. 1.8%). About 42% of patients receiving FOLFIRINOX also received G-CSF compared to only 5.3% in the gemcitabine group. This higher toxicity rate is the reason why this protocol is often modified including dropping the 5-FU bolus, reducing irinotecan and the 5-FU bolus, or reducing oxaliplatin. Interestingly, despite lower toxicity, survival benefits are comparable with the original FOLFIRINOX protocol in a meta-analysis [21, 22]. In addition, modified FOLFIRINOX (here, modified by omitting the 5-FU bolus) showed an impressive mOS of 14.4 months in the control arm of the SWOG 1313 trial (mFOLFIRINOX vs. mFOLFIRINOX + PEGPH20) [23]. This could be related to the therapy in further lines.

Of note, despite the higher rate of toxicity of the FOLFIRINOX protocol, deterioration of quality of life was significantly delayed in the FOLFIRINOX group of the phase III PRODIGE 4 Intergroup trial [12].

The PRODIGE 4 Intergroup trial only included patients up to the age of 75 years. Thus, there are no data from this prospective randomized trial for this group of patients regarding FOLFIRINOX efficacy and tolerability. Retrospective data suggest that modified FOLFIRINOX protocols have similar toxicity but also similar efficacy when compared to younger patients [22].

### Other 5-FU-Based Combination Treatments

Several phase III trials examined the effect of other 5-FU-based combination chemotherapies. Neither 5-FU plus mitomycin C [24] nor the combination of 5-FU, gemcitabine, epirubicin, and cisplatin [25, 26] has shown sufficient efficacy to qualify as a therapeutic standard.

The FIRGEM regime examined FOLFIRI.3 alternating with fixed-dose-rate gemcitabine compared to fixed-dose-rate gemcitabine alone in a randomized phase II trial [27]. The combination was superior to gemcitabine alone with respect to mOS (11 months vs. 8.2 months; HR = 0.71). However, due to a lack of phase III data, this combination cannot be regarded as a clinical standard. Another phase II trial examined the combination of 5-FU/leucovorin with nab-paclitaxel compared to gemcitabine plus nab-paclitaxel. The 4-month PFS as the primary endpoint of the trial was 56% for 5-FU/leucovorin plus nab-paclitaxel compared to 54% in the gemcitabine plus nab-paclitaxel group. The respective figures for mOS were 11.4 months vs. 9.2 months, respectively [28]. Thus, this protocol may be employed when gemcitabine cannot be used, e.g., in the case of gemcitabine intolerance.

### Targeted Therapies

The combination of gemcitabine with targeted therapies has so far not shown a clinically relevant survival benefit for patients with mPDAC. An exemption is the combination of gemcitabine with erlotinib, an EGFR tyrosine kinase inhibitor. There was a significant improvement in overall survival in favor of the combination (HR 0.82, *p* = 0.038). However, in the overall population, the difference in mOS between the groups was just 10 days. According to unplanned subgroup analysis, the difference in mOS was only more pronounced in the subgroup of patients developing a skin rash ≥ grade 2 in response to erlotinib (mOS 10.5 months vs. 5.8 months). Thus, if employed, this combination should not be continued when there is no rash during the first 8 weeks of treatment [29, 30].

## Second-Line and Further-Line Treatments

Upon progress under a first-line treatment, patients should receive a second-line treatment if their ECOG is ≤ 2. The majority of second-line treatments have been examined in patients receiving only gemcitabine as a first-line treatment (see Table 2).

**Table 2:** Metastatic pancreatic ductal adenocarcinoma: palliative second-line regimens

Regiment	Phase	*n*	mPFS (months)	mOS (months)	DCR (%)	References
Second-line therapy (mostly after gemcitabine mono in first-line treatment)						
Nal-Iri/5-FU/LV (NAPOLI-1 trial)	III	236	3.1	6.1	n.a	[33]
5-FU/LV			1.5	4.2		
OFF (CONKO-003 trial)	III	160	2.9	5.9	n.a	[35]
5-FU/LV			2.0	3.3		
mFOLFOX6, (PANCREOX trial)	III	108	3.1	6.1	44.7	[37]
5-FU/LV			2.9	9.9	55.3	
Gemcitabine/nab-paclitaxel (after FOLFIRINOX)	Cohort	57	5.1	8.8	58	[39]

*mPFS* median progression-free survival, *mOS* median overall survival, *DCR* disease control rate, *OFF* oxaliplatin, leucovorin, 5-FU, *LV* leucovorin, *5-FU* 5-FU, *nal-Iri* nanoliposomal irinotecan, *FOLFIRI* 5-FU, leucovorin, irinotecan, *FOLFOX* 5-FU, leucovorin, oxaliplatin

The three-arm, phase III NAPOLI study examined the combination of nanoliposomal irinotecan (nal-Iri) plus 5-FU/FA, nal-Iri alone, and 5-FU/FA alone in gemcitabine-pretreated patients with mPDAC and a Karnofsky performance status of ≥ 70%. The combination of nal-iri/5-FU/FA significantly improved overall survival compared to 5-FU/FA alone (6.2 months vs. 4.2 months; HR 0.75; *p* = 0.039) [33]. Nal-Iri alone was not superior to 5-FU/FA. The most frequent ≥ grade 3 side effects of the combination were neutropenia (27%), diarrhea (13%), emesis (11%), and fatigue (14%). Health-related quality of life was comparable between the combination and 5-FU/FA [34].

Another option in the second-line setting is the OFF regimen consisting of 5-FU/LV and oxaliplatin. This regimen can be employed in patients with progress under gemcitabine, an ECOG of ≤ 2 and polyneuropathy ≤ 2. This regimen improved OS (5.9 vs. 3.3 months; HR 0.66; *p* = 0.010) and time to tumor progression (2.9 months vs. 2 months; HR 0.68; *p* = 0.019) compared to 5-FU/LV alone [35, 36]. The PANCREOX trial compared a modified FOLFOX6 (mFOLFOX6) protocol with 5-FU/LV. mFOLFOX6 has a higher-dose intensity and worse tolerability compared to OFF; 20% of patients in the mFOLFOX6 arm compared to 2% in the 5-FU/LV arm stopped the treatment due to side effects. The combination did not improve mPFS as the primary endpoint of the trial (mFOLFOX6: 3.1 months, 5-FU/LV: 2.9 months; *p* = 0.99). OS was even shorter in the mFOLFOX6 arm (6.1 months vs. 9.9 months; *p* = 0.02). This may be explained by the high rate of post-progression treatment in the 5-FU/LV arm (FU/LV: 25% vs. mFOLFOX6: 7%; *p* = 0.015) [37]. However, even with a high rate of post-progression therapy, an mOS of 9.9 months achieved with only 5-FU/LV in a second-line setting is difficult to explain when the optimal mOS with FOLFIRIOX in the first line is only 11 months.

Despite the fact that there are no data from prospective, randomized trials for the second-line treatment after gemcitabine plus nab-paclitaxel in the first line, 5-FU/FA plus nal-Iri or OFF is a therapeutic option in the second-line setting since its components have not been employed in the first line. However, oxaliplatin should only be employed when there is no ≥ 2 grade residual polyneuropathy from nab-paclitaxel.

The situation after FOLFIRINOX is more complex since the components of the NAPOLI-1 trial (nal-Iri, %-FU, LV) as well as the OFF (oxaliplatin, 5-FU, LV) protocol are also parts of the FOLFIRINOX protocol. There are no prospective, randomized trials evaluating a second-line treatment after first-line FOLFIRINOX. In the PRODIGE 4 Intergroup study, 47% of patients received second-line treatment, mainly gemcitabine (82.5%) or gemcitabine-based combinations (12.5%). The combination of gemcitabine plus nab-paclitaxel as second-line treatment after FOLFIRONOX has only been examined in retrospective analyses and small cohort studies [38]. The efficacy of gemcitabine plus nab-paclitaxel in the second-line setting may be high with an mOS of 8.8 months and an mPFS of 5.1 months in a small cohort study. However, the toxicity of the combination was also high, with a grade 3/4 toxicity of about 40% of the patients consisting of neutropenia (12.5%), neurotoxicity (12.5%), asthenia (9%), and thrombocytopenia (6.5%) [39]. Thus, if employed, this treatment should only be offered to patients with an ECOG of 0–1.

After the failure of second-line treatment, there is very few data suggesting the benefit of a third-line treatment and no large, randomized trials are available. In the NAPOLI trial, about 30% of patients had received more than one previous chemotherapy. The combination of 5-FU/FA plus nal-Iri was efficacious also in this setting, given the patients had not received irinotecan previously (no previous irinotecan: HR 0.62; previous irinotecan: HR 1.25). Thus, this combination can also be used in lines beyond the second line when irinotecan has not been used during the previous lines of treatment [33].

### Therapeutic Options in Molecular Subgroups

Pancreatic cancer is characterized by high genomic heterogeneity. There are few subgroups that allow specific approaches. Recently, it could be shown that about 4–7% of an unselected Caucasian population exhibit germline mutation in the BRCA1 or -2 genes even without a clear family history [31, 40]. These tumors show a disturbed DNA homologous recombination leading to deficient repair of DNA double strand breaks. The tumors appear to be particularly sensitive to DNA crosslinking agents such as cisplatin or DNA repair inhibitors such as gemcitabine. This could be demonstrated in preclinical studies, but also in phase II trials examining the effect of gemcitabine plus cisplatin in mPDAC patients with a germline BRCA or PALB2 mutation [41].

The phase III POLO trial [31] examined maintenance treatment with the PARP inhibitor olaparib compared to placebo in patients with mPDAC and a germline mutation in BRCA1 or -2. Patients had to have at least stable disease under at least 16 weeks of a platinum-based first-line therapy; 81% of patients in the trial received FOLFIRINOX. In the overall population, mOS was about 19 months, stressing the beneficial role of a platinum-based treatment in patients with germ line BRCA1/2 mutations. Olaparib maintenance treatment significantly prolonged PFS, the primary endpoint of the study, compared to placebo (7.4 months vs. 3.8 months; HR 0.53; *p* = 0.004). However, there was no difference in overall survival between the two treatment arms (19 months vs. 19.2 months; HR 0.83, 95% CI 0.56–1.22; *p* = 0.3487) (see Table 1) [32]. Olaparib was well tolerated with anemia and fatigue as the most frequent ≥ grade 3 side effects.

Thus, patients with mPDAC should undergo testing for a germline BRCA1/2 mutation early after diagnosis since in these patients, a platinum-based treatment appears highly efficacious. At the moment, there is no direct comparison of whether cisplatin-based or oxaliplatin-based regimens are preferable in this situation[42]. Olaparib maintenance treatment is an interesting option for these specific patients. In case of proof of evidence of a BRCA-1/-2 germ line mutation, patients must be offered genetic counseling.

### Immunotherapy

Immune checkpoint inhibitors like PD1/PD-L1 mAbs are particularly efficacious in tumors with a deficient mismatch repair system (dMMR) or high microsatellite instability (MSI-H) [43]. The frequency of dMMR pancreatic cancers is only about 1% [44, 45]. There are few data on the use of checkpoint inhibitors in MSI-H/dMMR mPDAC. In a cohort of 22 MSI-H patients with advanced PDAC, mPFS under treatment with pembrolizumab was 2.1 months and mOS was 4 months [46]. In patients responding to the treatment, the duration of response was long, with 13.4 months. Thus, unfortunately, only a subgroup of the patient with MSI-H /dMMR, but not the whole group, appears to benefit substantially from a checkpoint inhibitor treatment. So far, there is no evidence that checkpoint inhibitors have any benefit at all in PDACs without dMMR/MSI-H status.

### Next Steps

Pancreatic cancer remains a difficult treatment area. However, identifying appropriate subgroups of pancreatic cancers may offer the chance to substantially improve outcomes. Such subgroups are tumors with somatic DNA damage repair deficiencies such as ATM [47, 48]. Also, the subgroup of KRAS wild-type tumors is interesting, exhibiting targetable fusions such as NRG1 fusions [49]. Another albeit very small subgroup PDACs with NTRK fusions that are also targetable. In addition, novel predictive screening approaches such as tumor–organoid pharmacotyping and treatment selection may also help to select a more efficacious treatment for an individual tumor [50]. Finally, the future potential availability of selective inhibitors of mutated KRAS could also mean substantial progress in PDAC treatment.
